# The effect gap of hypertension health management services in poverty and non-poverty counties on the hypertension control: evidence from China Chronic Diseases Risk Factors Surveillance

**DOI:** 10.1186/s41043-023-00380-8

**Published:** 2023-05-02

**Authors:** Bo Jiang, Limin Wang, Mei Zhang, Zhenping Zhao, Xiao Zhang, Chun Li, Maigeng Zhou

**Affiliations:** National Center for Chronic and Non-communicable Disease Control and Prevention, Chinese Center for Disease Control and Prevention, Beijing, China

**Keywords:** Hypertension, Essential public health services, Health poverty alleviation project

## Abstract

**Background:**

The Chinese government implemented the health poverty alleviation project (HPAP) since 2016 in poverty counties (PCs). To evaluate the effect of the HPAP on hypertension health management and control in PCs is vital for the policy improvement.

**Methods:**

China Chronic Disease and Risk Factors Surveillance programme were conducted from August 2018 to June 2019. A total of 95,414 participants aged 35 and above from 59 PCs and 129 non-poverty counties (NPCs) were involved in this study. Hypertension prevalence, hypertension control, treatment and health management prevalence, and physical examination proportion were calculated and compared by PCs and NPCs. Logistic regression was employed to explore the association between hypertension control and management services.

**Results:**

The hypertension prevalence in NPCs was significantly higher than that in PCs (NPCs 46.1% vs. PCs 41.2%, *P* < 0.001). The NPCs participants had a higher hypertension control prevalence (NPCs 32.7% vs. PCs 27.3%,* P* < 0.001) and treatment prevalence (NPCs 86.0% vs. PCs 80.0%,* P* < 0.001) than that in PCs. The proportion of physical examination in one year in NPCs was significantly higher than that in PCs (NPCs 37.0% vs. PCs 29.5%, *P* < 0.001). The proportion of diagnosed hypertension patients without hypertension health management in NPCs was significantly higher than that in PCs (NPCs 35.7% vs. PCs 38.4%,* P* < 0.001). Multivariable logistic regression showed that standardized and non-standardized hypertension health management were positively correlated with hypertension control in NPCs, and standardized hypertension health management was positively correlated with hypertension control in PCs.

**Conclusions:**

These findings show the equity and accessibility gap of health resources still existed between PCs and NPCs under the influence of the HPAP. Hypertensive health management was effective for hypertension control in both PCs and NPCs. However, the quality of management services still needs to be improved.

## Background

Ending poverty is the primary goal of the United Nations (UN) 2030 Agenda for Sustainable Development, and it is also one of the main tasks of China’s social and economic development [1]. By 2020, 98.99 million poverty people in China have been lifted out of poverty, and absolute poverty has been eliminated [2]. The poverty reduction goal of the UN 2030 Agenda for Sustainable Development has been achieved ten years ahead of schedule in China. Continuously consolidating and expanding the achievements of poverty alleviation in China is the next stage task. There is a vicious cycle between disease and poverty. Poverty causes illness and death by affecting the population’s nutrition, education, lifestyle, medical equity and access. The poverty population likely loses the opportunity for medical security, medical care, and essential public health services [3]. The loss of the workforce, retirement, or die prematurely as the aggravation of illness leads to lower income and higher financial burden, further reducing access to health services for the poverty population [4–6]. People with higher incomes have better health, and vice versa. Poverty and health adversities create more severe social problems through accumulation across generations. Hence, health poverty alleviation is an essential part of poverty alleviation policies.

In 2015, the “Decision of the Central Committee of the Communist Party of China and the State Council on Winning the Tough Battle against Poverty” proposed alleviating poverty for more than 70 million rural poverty populations by 2020 [7]. Following this policy, various state departments issued 118 relevant policy documents and implementation plans [8]. The Chinese government implemented the health poverty alleviation project (HPAP) as an essential part of the poverty alleviation policy since 2016 [9, 10]. The policies included the guarantee of essential medical and health services for poverty people in PCs, the improvement of public health, the improvement of medical institution services, and the optimization of medical resource allocation [11].

The HPAP framework required counties to strengthen chronic diseases prevention and control by improving public health services and health management. Essential public health services (EPHS), a series of community-based management programs, were one of the most important and effective measures to increase the prevention and treatment of infectious, endemic and chronic diseases in PCs [12]. Hypertension health management was one of the important parts of the EPHS, which provided management services for hypertensive patients aged 35 and above [13]. Hypertension health management was a significant and effective measure for hypertension control in China [14, 15]. The HPAP required improving the quality of EPHS, especially for hypertension patients in PCs.

As a critical chronic disease, hypertension is a cardiovascular disease and a risk factor for other diseases. Hypertension causes a considerable burden of disease in China, and it is also an essential part of chronic disease prevention and control in the HPAP and EPHS [16, 17]. After implementing the HPAP for several years, the current effect evaluation studies pay more attention to the financial risk protection of the poverty population [8, 18] and the effect of the medical insurance system [19–21]. And hypertension health management studies rarely consider the difference between PCs and NPCs [14, 15]. The contrast of hypertension management and control effect between PCs with the HPAP and NPCs needs further attention. Therefore, this paper aims to provide evidence for the effect gap of hypertension management and control in PCs with the HPAP and NPCs by using the latest national surveillance data of chronic diseases. This study assesses the effect of the HPAP on hypertension management and control by analyzing the control, treatment, and management of hypertension patients in PCs.

## Methods

### Data source and sampling method

We used data from the China Chronic Disease and Risk Factors Surveillance (CCDRFS) programme conducted from August 2018 to June 2019 according to a standard protocol. The CCDRFS was used to evaluate the prevalence of major chronic diseases and the associated behavioral and metabolic risk factors. The multistage stratified sampling method was used to sample, and the protocol for collecting the CCDRFS data has been described elsewhere [22–24]. The representative survey collected data from 298 counties (districts) in 31 provinces (autonomous regions, municipalities) in China [25, 26]. A total of 184,876 participants (living at their current residence for at least six months within the year before the survey) aged 18 and above were investigated in the CCDRFS. All participants signed informed consent.

### Data collection

The surveillance included questionnaire investigation, medical examination, and laboratory tests. All these contents followed a unified and standard protocol for each site. Demographic characteristics, prevalence, treatment and control of major chronic diseases, health management services, socioeconomic and behavioral factors information of participants were collected by trained staff through questionnaire investigation. Blood pressure, height, and weight were measured by trained staff with standard tools. A digital blood pressure monitor (OMRON HBP1300) was used to measure systolic blood pressure (SBP) and diastolic blood pressure (DBP) three times, respectively. The averages of SBP and DBP were calculated for analysis. The laboratory tests included blood and urine sample tests. Blood sample tests contained fasting blood glucose(FBG), blood glucose 2 h after taking 75 g oral glucose sugar(participants without self-reported diabetes history), total cholesterol (TC), high-density lipoprotein cholesterol (HDL-C), serum creatinine, and albumin. Blood samples were collected after at least 8 h fast from every participant and participants taking 75 g oral glucose sugar. Morning urination samples were collected to detect urine creatinine and microalbumin. Plasma glucose was measured by hexokinase method, TC was measured by cholesterol oxidase p-aminophenazone (CHOD-PAP) method, HDL-C was measured by homogeneous enzyme colorimetry, and serum creatinine and urine creatinine were measured by enzyme coupled creatine oxidase.

### Demographic characteristics

Demographic Characteristics comparisons were made between PCs and NPCs. Demographic included age, sex (male or female), education (< primary school, primary school, middle school, ≥ high school), marital status (married, single/widow/divorce/separated), medical insurance status (yes or no), location (urban or rural), and geographic region [eastern (Beijing, Tianjin, Hebei, Liaoning, Shanghai, Jiangsu, Zhejiang, Fujian, Shandong, Guangdong and Hainan), central (Shanxi, Inner Mongolia, Jilin, Heilongjiang, Anhui, Jiangxi, Henan, Hubei, Hunan, Guangxi), western (Sichuan, Guizhou, Yunnan, Tibet, Shaanxi, Gansu, Qinghai, Ningxia, Xinjiang)]. Household income per capita per year was converted into quartiles (≤ $727.8, $727.8 ~ 1455.5, $1455.5 ~ 2765.5, > $2765.5, unknown).The State Council had determined 832 national PCs and 334 deep PCs in China [27, 28]. The national PCs were determined according to the per capita county GDP (gross domestic product), per capita general budget income of county finance, per capita net income of county farmers, and other factors. The deep PCs were determined according to the poverty population scale, the economic development level, the difficulty of poverty alleviation, and other factors.

### Hypertension health management services

Diagnosed hypertension patients were divided into three groups (non-standardized, standardized or no) according to whether the patients participated in the hypertension health management services involved in the HPAP. The “standardized management” group included diagnosed hypertension patients participating in health management who have received at least four blood pressure measurements per year provided by doctors in primary medical and health institutions, as well as guidance and suggestions on medication, diet, physical activity, tobacco and alcohol control. The “non-standardized management” group included diagnosed hypertension patients without enough blood pressure measurements or guidance and suggestions on medication, diet, physical activity, tobacco and alcohol control. The “no” group included diagnosed patients without hypertension health management services.

### Influencing factors of hypertension control

Behavioral influencing factors of hypertension control contained smoking status (current smoker, former smoker, or nonsmoker), current alcohol use (within 30 days, out of 30 days, or nondrinker), low physical activity (< 150 min/week), low fruit and vegetable intake (< 400 g/d), high red meat intake (≥ 100 g/d), high salt intake (≥ 5 g/d), and high oil intake (≥ 25 g/d). Body-mass index (BMI) was calculated as weight (kg) divided by height (m) squared and divided into four groups (< 18.5 kg/m^2^, 18.5–25 kg/m^2^, 25–30 kg/m^2^ or > 30 kg/m^2^) according to WHO criteria. Diabetes were defined as participants with self-reported diabetes diagnosed by a health professional medical institution or with FBG ≥ 7 mmol/L or 2-h plasma glucose ≥ 11.1 mmol/L after an oral 75 g anhydrous glucose. The urine microalbumin to creatinine ratio (ACR) was calculated using urinary microalbumin divided by urinary creatinine. The glomerular filtration rate (eGFR) was calculated using the CKD-EPI equation [29]. Chronic kidney disease (CKD) was defined as eGFR < 60 mL/(min/1.73 m^2^) or ACR ≥ 30 mg/g according to the Clinical Practice Guideline for Evaluation and Management of CKD by Kidney Disease Improving Global Outcomes (KDIGO). Non-HDL-C was calculated by TC subtracting HDL-C and Non-HDL-C ≥ 4.90 mmol/L, defined as high Non-HDL-C [30].

### Health outcomes

Primary health outcomes were hypertension prevalence, hypertension control, and health management prevalence. The hypertension population was divided into two categories: diagnosed hypertension patients (self-reported hypertension diagnosed by a health professional medical institution) or new found population (SBP ≥ 140 mmHg or DBP ≥ 90 mmHg in this survey but not self-reported hypertension). Hypertension control was defined as SBP < 140 mmHg and DBP < 90 mmHg among diagnosed patients under 65 years old and SBP < 150 mmHg and DBP < 90 mmHg among diagnosed patients over 65 years old. Hypertension control prevalence was only calculated in diagnosed patients.

Secondary outcomes were physical examination proportion and hypertension treatment prevalence. Physical examination was collected through the question “When was your last physical examination?” in the questionnaire and divided into three categories: one year and below, more than one year, or no. The hypertension treatment was defined as the diagnosed hypertension patients using antihypertensive drugs. Hypertension treatment prevalence was only calculated among diagnosed patients.

### Statistical analysis

We used count and proportions to describe qualitative data, and *χ*^2^ or Wilcoxon rank sum tests to examine differences in categorical variables. Hypertension control prevalence was calculated separately by sex (men vs. women) and locality of residence (urban vs. rural). Before multivariate analysis, we examined differences in influencing factors between controlled and uncontrolled hypertension patients by PCs and NPCs, respectively. We used a multivariate logistic regression model to explore the association between hypertension control and health management services. Models were built in NPCs (*n* = 14,497) and PCs (*n* = 4932), respectively. Only diagnosed hypertension patients were involved in regression model analysis. *P* values were two sided and *P* < 0.05 was considered statistically significant. The analyses were calculated using SAS ver. 9.4 (SAS institute).

## Results

### Demographic characteristics and influencing factors

**Fig. 1:**
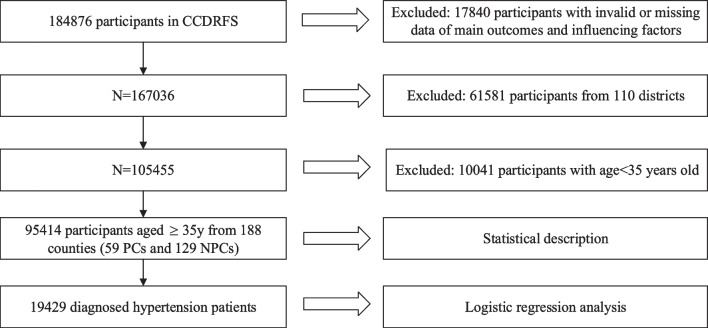
Flowchart of study population

A total of 17,840 participants were excluded because of the incomplete main outcome, demographic, and influencing factors information. There were 6 districts and 59 counties from 298 surveillance counties included in 832 national PCs and 334 deep PCs. We considered districts had a high level of industrialization, urbanization, and modernization. District residents were more likely to obtain better economic, medical, environmental, and transportation resources. So 61,581 participants from 110 districts were excluded. We also excluded 10,041 participants younger than 35 years old because the target population of hypertension health management services was people aged 35 years and above. Finally, 95,414 participants aged 35 and above from 188 counties (59 PCs and 129 NPCs) were involved in this study (Fig. 1). The mean age was 57.6 ± 11.1. Table 1 showed the demographic characteristics and influencing factors of participants in PCs and NPCs. PCs included 28,820 participants, and the mean age was 58.2 ± 10.9. NPCs included 66,594 participants, and the mean age was 56.31 ± 11.32. The differences in the proportion of age, sex, education, location, geographic region, marital status and medical insurance status groups were statistically significant between PCs and NPCs. The hypertension prevalence in NPCs was significantly higher than that in PCs (NPCs 46.1% vs. PCs 41.2%, *P* < 0.001). The diagnosed hypertension prevalence was significantly higher than that in PCs (NPCs 21.8% vs. PCs 17.1%, *P* < 0.001). The NPCs participants had a higher hypertension control prevalence (NPCs 32.7% vs. PCs 27.3%,* P* < 0.001) and treatment prevalence (NPCs 86.0% vs. PCs 80.0%,* P* < 0.001) than that in PCs. The proportion of physical examination in one year in NPCs was significantly higher than that in PCs (NPCs 37.0% vs. PCs 29.5%, *P* < 0.001).

**Table 1:** Characteristics of the population aged 35 and above

Characteristics	Total (*n* = 95,414)	NPCs (*n* = 66,594)	PCs (*n* = 28,820)	*χ*^2^/*Z*	*P*
*Age*					
35–49	25,632 (26.9)	16,422 (24.7)	9210 (32.0)	− 23.0062^†^	< 0.001
50–59	28,815 (30.2)	20,170 (30.3)	8645 (30.0)		
60–69	27,522 (28.8)	20,284 (30.4)	7238 (25.1)		
70 and above	13,445 (14.1)	9718 (14.6)	3727 (12.9)		
*Sex*					
Male	43,483 (45.6)	30,210 (45.4)	13,273 (46.0)	3.8651	0.049
Female	51,931 (54.4)	36,384 (54.6)	15,547 (54.0)		
*Education*					
Below primary school	36,674 (38.4)	22,657 (34.0)	14,017 (48.6)	− 45.0893^†^	< 0.001
Primary school	22,553 (23.6)	15,989 (24.0)	6564 (22.8)		
Middle school	26,184 (27.5)	20,125 (30.2)	6059 (21.0)		
High school or higher	10,003 (10.5)	7823 (11.8)	2180 (7.6)		
*Marital status*					
Married	88,221 (92.5)	61,805 (92.8)	26,416 (91.7)	38.1701	< 0.001
Single/Widow/Divorce/Separated	7193 (7.5)	4789 (7.2)	2404 (8.3)		
*Medical insurance status*					
No	1475 (1.6)	1144 (1.7)	331 (1.1)	42.8434	< 0.001
Yes	93,939 (98.4)	65,450 (98.3)	28,489 (98.9)		
*Physical examination*					
One year and below	33,142 (34.7)	24,631 (37.0)	8511 (29.5)	29.2118^†^	< 0.001
More than one year	10,116 (10.6)	7784 (11.7)	2332 (8.1)		
No	52,156 (54.7)	34,179 (51.3)	17,977 (62.4)		
*Location*					
Urban	25,203 (26.4)	19,677 (29.6)	5526 (19.2)	1113.6180	< 0.001
Rural	70,211 (73.6)	46,917 (70.4)	23,294 (80.8)		
*Geographic region*					
Eastern	31,174 (32.7)	28,602 (42.9)	2572 (8.9)	12,700.9900	< 0.001
Central	27,163 (28.5)	18,708 (28.1)	8455 (29.3)		
Western	37,077 (38.8)	19,284 (29.0)	17,793 (61.8)		
*Household income per capita per year*					
> $2765.5	18,246 (19.1)	14,704 (22.1)	3542 (12.3)	2179.2643^†^	< 0.001
$1455.5–2765.5	14,661 (15.4)	11,213 (16.8)	3448 (12.0)		
$727.8–1455.5	18,111 (19.0)	12,437 (18.7)	5674 (19.7)		
≤ $727.8	22,123 (23.2)	13,753 (20.7)	8370 (29.0)		
Unknown	22,273 (23.3)	14,487 (21.7)	7786 (27.0)		
*Hypertension*					
Diagnosed	19,429 (20.4)	14,497 (21.8)	4932 (17.1)	16.1578	< 0.001
New discovered	23,137 (24.2)	16,180 (24.3)	6957 (24.1)		
No	52,848 (55.4)	35,917 (53.9)	16,931 (58.8)		
*Control of diagnosed hypertension patients* (*n* = 19,429)^*^					
No	13,339 (68.7)	9752 (67.3)	3587 (72.7)	50.9801	< 0.001
Yes	6090 (31.3)	4745 (32.7)	1345 (27.3)		
*Treatment of diagnosed hypertension patients* (*n* = 19,429)^*^					
No	3023 (15.6)	2035 (14.0)	988 (20.0)	100.6693	< 0.001
Yes	16,406 (84.4)	12,462 (86.0)	3944 (80.0)		
*Diabetes*					
No	79,433 (83.3)	54,385 (81.7)	25,048 (86.9)	396.9038	< 0.001
Yes	15,981 (16.7)	12,209 (18.3)	3772 (13.1)		
*High non-HDL-C*					
No	85,563 (89.7)	59,360 (89.1)	26,203 (90.9)	69.0170	< 0.001
Yes	9851 (10.3)	7234 (10.9)	2617 (9.1)		
*Chronic kidney disease*					
No	83,509 (87.5)	58,435 (87.7)	25,074 (87.0)	10.2525	0.0014
Yes	11,905 (12.5)	8159 (12.3)	3746 (13.0)		
*BMI(kg/m*^*2*^*)*					
< 18.5	2826 (3.0)	1840 (2.8)	986 (3.4)	− 16.7724^†^	< 0.001
18.5–25	52,892 (55.4)	35,916 (53.9)	16,976 (58.9)		
25–30	33,319 (34.9)	24,104 (36.2)	9215 (32.0)		
≥ 30	6377 (6.7)	4734 (7.1)	1643 (5.7)		
*Smoking status*					
Current smoker	64,159 (67.3)	44,589 (66.9)	19,570 (67.9)	98.0768	< 0.001
Quit smoker	6531 (6.8)	4911 (7.4)	1620 (5.6)		
Nonsmoker	24,724 (25.9)	17,094 (25.7)	7630 (26.5)		
*Current alcohol use*					
No	63,428 (66.5)	43,462 (65.3)	19,966 (69.3)	−11.7240^†^	< 0.001
Out of 30 days	7967 (8.3)	5792 (8.7)	2175 (7.5)		
Within 30 days	24,019 (25.2)	17,340 (26.0)	6679 (23.2)		
*Low physical activity*					
Yes	77,201 (80.9)	54,063 (81.2)	23,138 (80.3)	10.5133	0.001
No	18,213 (19.1)	12,531 (18.8)	5682 (19.7)		
*Low vegetable and fruit intake*					
Yes	46,296 (48.5)	34,470 (51.8)	11,826 (41.0)	926.7173	< 0.001
No	49,118 (51.5)	32,124 (48.2)	16,994 (59.0)		
*High red meat intake*					
Yes	74,341 (77.9)	52,555 (78.9)	21,786 (75.6)	129.2461	< 0.001
No	21,073 (22.1)	14,039 (21.1)	7034 (24.4)		
*High salt intake*					
Yes	55,138 (57.8)	36,983 (55.6)	18,155 (63.0)	490.0296	< 0.001
No	33,357 (35.0)	24,330 (36.5)	9027 (31.3)		
Unknown	6919 (7.2)	5281 (7.9)	1638 (5.7)		
*High oil intake*					
Yes	66,273 (69.4)	47,044 (70.6)	19,229 (66.7)	167.6646	< 0.001
No	24,593 (25.8)	16,366 (24.6)	8227 (28.6)		
Unknown	4548 (4.8)	3184 (4.8)	1364 (4.7)		

Data are presented as *n* (%)

*Hypertension control and treatment prevalence are only calculated in diagnosed hypertension patients

^†^Wilcoxon rank sum tests

### Health management services situation

**Fig. 2:**
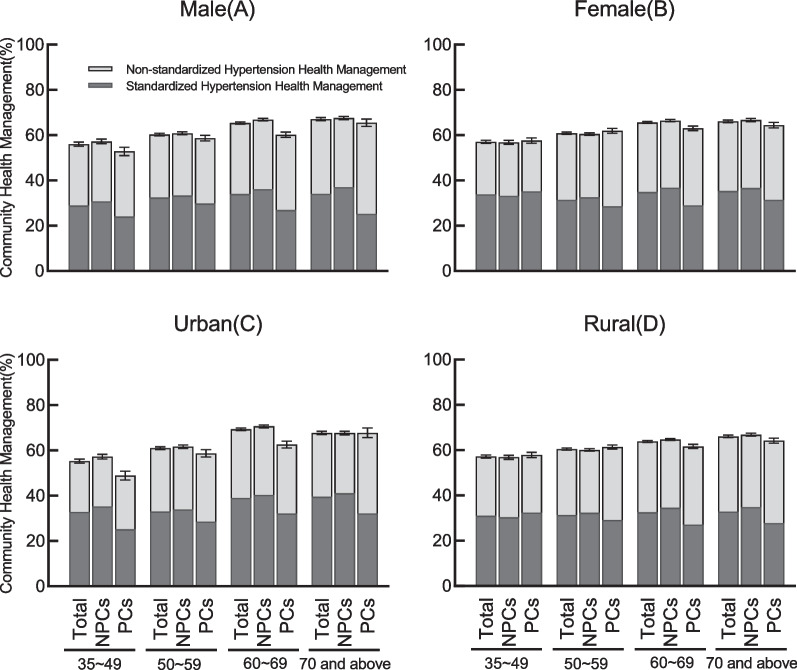
Hypertension health management of diagnosed hypertension patients aged 35 and above, by sex (**A**, **B**) and location (**C**, **D**) locality

Among all diagnosed hypertension patients, the hypertension health management prevalence was 63.6%, and the standardized and non-standardized hypertension health management prevalence was 33.6% and 30.1%, respectively. Figure 2 showed hypertension health management prevalence in NPCs and PCs by sex (men vs. women) and locality of residence (urban vs. rural). The proportion of diagnosed hypertension patients without hypertension health management in PCs was significantly higher than that in NPCs (NPCs 35.7% vs. PCs 38.4%,* P* < 0.001). Table 2 showed the proportion of patients with more than four times/year blood pressure measurement (NPCs 75.8% vs. PCs 69.8%, *P* < 0.001), medication guidance (NPCs 84.09% vs. PCs 80.6%, *P* < 0.001) in NPCs was significantly higher than that in PCs. The proportion of patients with guidance of dietary (NPCs 78.8% vs. PCs 77.1%, *P* = 0.049), physical activity (NPCs 73.1% vs. PCs 67.1%, *P* < 0.001), tobacco control (NPCs 22.7% vs. 21.4% PCs, *P* < 0.001), and alcohol control (NPCs 33.3% vs. PCs 28.1%, *P* < 0.001) in NPCs was also higher than that in PCs.

**Table 2:** Details of hypertension health management for diagnosed hypertension patients in NPCs and PCs

Characteristics	NPCs	PCs	*χ*^2^/*Z*	*P*
*Hypertension health management*				
Yes (Standardized)	5099 (35.2)	1419 (28.8)	68.9629	< 0.001
Yes (Non-standardized)	4222 (29.1)	1617 (32.8)		
No	5176 (35.7)	1896 (38.4)		
Blood pressure measurement*				
Four times or above	7072 (75.9)	2119 (69.8)	6.2847^†^	< 0.001
One-three times	1515 (16.2)	654 (21.5)		
No	734 (7.9)	263 (8.7)		
Medication guidance management*				
Yes	7829 (84.0)	2448 (80.6)	18.4762	< 0.001
No	1492 (16.0)	588 (19.4)		
Dietary guidance management*				
Yes	7348 (78.8)	2342 (77.1)	3.8727	0.049
No	1973 (21.2)	694 (22.9)		
Physical activity guidance management*				
Yes	6816 (73.1)	2037 (67.1)	40.9927	< 0.001
No	2505 (26.9)	999 (32.9)		
Tobacco control guidance management*				
Yes	2112 (22.7)	650 (21.4)	20.1609	< 0.001
No	979 (10.5)	408 (13.4)		
Nonsmoker	6230 (66.8)	1978 (65.2)		
Alcohol control guidance management*				
Yes	3107 (33.3)	852 (28.1)	29.6919	< 0.001
No	1168 (12.5)	425 (14.0)		
Non-drinker	5046 (54.2)	1759 (57.9)		

Data are presented as *n* (%)

*Calculate only in hypertension patients with hypertension health management

^†^Wilcoxon rank sum tests

### Influencing factors associated with hypertension control

Table 3 showed the different hypertension control prevalence by influencing factors between NPCs and PCs. In NPCs, there were significant differences for the hypertension control prevalence in diabetes, high non-HDL-C, CKD, smoking status, current alcohol status, BMI and hypertension health management. In PCs, the significant difference in the proportions of hypertension control was in CKD, high non-HDL-C, current alcohol status, BMI and hypertension health management. Table 4 showed multivariate logistic regression results by PCs and NPCs, respectively. In NPCs, rural, central, western, diabetes, high non-HDL-C, CKD, BMI with 25 ~ 30 kg/m^2^ and ≥ 30 kg/m^2^, former smokers, alcohol use within and out of 30 days were negatively correlated with hypertension control; 60 ~ 69 and ≥ 70 age groups, household income per capita per year of $727.8 to $1455.5 and $1455.5 to $2765.5, standardized and non-standardized hypertension health management were positively correlated with hypertension control. In PCs, CKD, BMI with 25–30 kg/m^2^ and ≥ 30 kg/m^2^, and alcohol use within 30 days were negatively correlated with hypertension control; in 60–69 and ≥ 70 age groups, high school or higher, and standardized hypertension health management were positively correlated with hypertension control.

**Table 3:** Influence factors of hypertension control among the diagnosed hypertension patients in NPCs and PCs

Influence factors	NPCs		PCs	
	Uncontrolled	Controlled	Uncontrolled	Controlled
*Diabetes*				
No	6656 (66.5)	3359 (33.5)	2738 (72.8)	1023 (27.2)
Yes	3096 (69.1)	1386 (30.9)	849 (72.5)	322 (27.5)
*χ*^2^	9.6238		0.0399	
*P*	0.002		0.842	
*High non-HDL-C*				
No	8361 (66.5)	4204 (33.5)	3131 (72.2)	1204 (27.8)
Yes	1391 (72.0)	541 (28.0)	456 (76.4)	141 (23.6)
*χ*^2^	22.6391		4.5693	
*P*	< 0.001		0.033	
*Chronic kidney disease*				
No	7408 (65.8)	3847 (34.2)	2638 (70.9)	1081 (29.1)
Yes	2344 (72.3)	898 (27.7)	949 (78.2)	264 (21.8)
*χ*^2^	48.0227		24.5939	
*P*	< 0.001		< 0.001	
*BMI(kg/m*^*2*^*)*				
< 18.5	89 (57.4)	66 (42.6)	63 (67.0)	31 (33.0)
18.5–25	3822 (64.7)	2089 (35.3)	1575 (69.6)	687 (30.4)
25–30	4613 (68.7)	2105 (31.3)	1551 (74.9)	521 (25.1)
≥ 30	1228 (71.7)	485 (28.3)	398 (79.0)	106 (21.0)
*χ*^2^	46.2596		27.1213	
*P*	< 0.001		< 0.001	
*Smoking status*				
Current smoker	2035 (65.6)	1067 (34.4)	773 (71.5)	308 (28.5)
Quit smoker	902 (63.5)	519 (36.5)	253 (70.1)	108 (29.9)
Nonsmoker	6815 (68.3)	3159 (31.7)	2561 (73.4)	929 (26.6)
*χ*^2^	18.2711		2.8352	
*P*	< 0.001		0.242	
*Current alcohol use*				
No	6777 (66.2)	3463 (33.8)	2683 (71.8)	1056 (28.2)
Within 30 days	2247 (70.2)	953 (29.8)	672 (76.5)	206 (23.5)
Out of 30 days	728 (68.9)	329 (31.1)	232 (73.7)	83 (26.3)
*χ*^2^	19.3816		8.3366	
*P*	< 0.001		0.016	
*Low physical activity*				
No	7697 (67.4)	3720 (32.6)	2830 (73.1)	1043 (26.9)
Yes	2055 (66.7)	1025 (33.3)	757 (71.5)	302 (28.5)
*χ*^2^	0.5340		1.0566	
*P*	0.465		0.304	
*Low vegetable and fruit intake*				
No	4906 (66.6)	2456 (33.4)	1410 (72.8)	527 (27.2)
Yes	4846 (67.9)	2289 (32.1)	2177 (72.7)	818 (27.3)
*χ*^2^	2.6929		0.0066	
*P*	0.101		0.936	
*High red meat intake*				
No	8169 (67.5)	3942 (32.5)	2923 (72.2)	1123 (27.8)
Yes	1583 (66.3)	803 (33.7)	664 (74.9)	222 (25.1)
*χ*^2^	1.1069		2.6703	
*P*	0.293		0.102	
*High salt intake*				
No	3435 (66.6)	1721 (33.4)	1078 (74.7)	365 (25.3)
Yes	5507 (67.8)	2612 (32.2)	2329 (72.2)	897 (27.8)
Unknown	810 (66.3)	412 (33.7)	180 (68.4)	83 (31.6)
*χ*^2^	2.6742		5.7446	
*P*	0.263		0.057	
*High oil intake*				
No	2352 (65.7)	1229 (34.3)	1047 (72.7)	394 (27.3)
Yes	6927 (67.8)	3293 (32.2)	2377 (72.8)	890 (27.2)
Unknown	473 (68.0)	223 (32.0)	163 (72.8)	61 (27.2)
*χ*^2^	5.4642		0.0052	
*P*	0.065		0.997	
*Hypertension health management*				
Yes (Standardized)	3246 (63.7)	1853 (36.3)	990 (69.8)	429 (30.2)
Yes (Non-standardized)	2827 (67.0)	1395 (33.0)	1175 (72.7)	442 (27.3)
No	3679 (71.1)	1497 (28.9)	1422 (75.0)	474 (25.0)
*χ*^2^	64.4638		11.2085	
*P*	< 0.001		0.004	

Data are presented as *n* (%)

**Table 4:** *OR* (%95 *CI*) of hypertension control in NPCs and PCs according to the hypertension health management

Characteristics	NPCs	PCs
*Age*		
35–49	Ref	Ref
50–59	0.96 (0.83, 1.11)	0.96 (0.75, 1.23)
60–69	1.57 (1.36, 1.81)	1.63 (1.28, 2.07)
70 and above	2.21 (1.90, 2.58)	2.53 (1.95, 3.28)
*Sex*		
Male	Ref	Ref
Female	1.06 (0.95, 1.18)	1.00 (0.83, 1.21)
*Education*		
Below Primary school	Ref	Ref
Primary school	1.03 (0.94, 1.14)	1.19 (1.00, 1.41)
Middle school	1.07 (0.96, 1.18)	1.13 (0.92, 1.37)
High school or higher	1.04 (0.91, 1.20)	1.33 (1.00, 1.77)
*Marital status*		
Married	Ref	Ref
Single/Widow/Divorce/Separated	0.92 (0.81, 1.04)	0.84 (0.69, 1.02)
*Medical insurance status*		
Yes	Ref	Ref
No	1.19 (0.89, 1.60)	1.16 (0.59, 2.26)
*Location*		
Urban	Ref	Ref
Rural	0.81 (0.75, 0.88)	1.09 (0.93, 1.29)
*Geographic region*		
Eastern	Ref	Ref
Central	0.73 (0.67, 0.80)	1.08 (0.86, 1.35)
Western	0.87 (0.80, 0.95)	0.85 (0.68, 1.06)
*Household income per capita*		
≤ $727.8	Ref	Ref
$727.8–1455.5	1.38 (1.23, 1.54)	1.22 (0.97, 1.53)
$1455.5–2765.5	1.17 (1.04, 1.32)	1.14 (0.90, 1.44)
> $2765.5	1.03 (0.92, 1.15)	1.09 (0.90, 1.32)
Unknown	1.03 (0.92, 1.15)	0.96 (0.80, 1.14)
*Diabetes*		
No	Ref	Ref
Yes	0.87 (0.81, 0.95)	1.04 (0.89, 1.21)
*High non-HDL-C*		
No	Ref	Ref
Yes	0.82 (0.74, 0.92)	0.84 (0.68, 1.03)
*Chronic kidney disease*		
No	Ref	Ref
Yes	0.67 (0.61, 0.73)	0.61 (0.52, 0.71)
*BMI(kg/m*^*2*^*)*		
18.5–25	Ref	Ref
< 18.5	1.17 (0.84, 1.62)	1.09 (0.70, 1.72)
25–30	0.91 (0.84, 0.98)	0.85 (0.74, 0.98)
≥ 30	0.85 (0.75, 0.96)	0.72 (0.56, 0.91)
*Smoking status*		
Current smoker	Ref	Ref
Former smoker	0.77 (0.69, 0.87)	0.90 (0.74, 1.11)
Nonsmoker	0.97 (0.85, 1.12)	0.97 (0.74, 1.27)
*Current alcohol use*		
No	Ref	Ref
Within 30 days	0.75 (0.68, 0.83)	0.74 (0.61, 0.90)
Out of 30 days	0.85 (0.74, 0.98)	0.91 (0.69, 1.19)
*Low physical activity*		
No	Ref	Ref
Yes	0.99 (0.91, 1.08)	1.01 (0.86, 1.18)
*Low vegetable and fruit intake*		
No	Ref	Ref
Yes	0.95 (0.88, 1.02)	1.01 (0.86, 1.18)
*High red meat intake*		
No	Ref	Ref
Yes	1.05 (0.96, 1.16)	1.00 (0.83, 1.20)
*High salt intake*		
No	Ref	Ref
Yes	1.00 (0.93, 1.09)	1.13 (0.98, 1.31)
Unknown	1.11 (0.95, 1.31)	1.68 (1.17, 2.41)
*High oil intake*		
No	Ref	Ref
Yes	0.95 (0.87, 1.04)	0.94 (0.81, 1.09)
Unknown	0.96 (0.77, 1.19)	0.76 (0.51, 1.13)
*Hypertension health management*		
No	Ref	Ref
Yes (Standardized)	1.29 (1.18, 1.40)	1.35 (1.15, 1.58)
Yes (Non-standardized)	1.14 (1.04, 1.25)	1.13 (0.97, 1.32)

## Discussion

In this study, the result shows although PCs have a lower hypertension prevalence, there is still a gap in the proportion of physical examination in one year, the hypertension control and treatment prevalence, the hypertension health management and standardized management prevalence between PCs and NPCs. Multivariable logistic regression analysis shows that hypertension health management is a positive factor for hypertension control in PCs and NPCs. The coexistence of multiple chronic diseases in hypertension patients is another difficulty of hypertension control.

Our study shows that participants who live in PCs have a lower hypertension prevalence than those who live in NPCs. Significant economic differences between NPCs and PCs cause differences in residents’ education, medical, transportation, and lifestyle. It is believed that people in economically developed areas have a high hypertension prevalence because of adequate food, high life pressure, and unhealthy lifestyles, but more cardiovascular deaths occur in low and middle-income countries [31]. With improving the economy in PCs, more chronic diseases should become the major public health challenge. No matter the proportion of physical examination in 1 year, the hypertension control and treatment or hypertension health management in PCs are all lower than that in NPCs. It may be caused by the lack of medical resourcesand health service underutilization [12, 32]. There is a gap between the accessibility and equity of health resources among different provinces in China [33]. PCs need more medical and public health service resources, including human and material resources [12]. The HPAP is committed to solving the problem of poverty and healthcare equity and access. For chronic diseases, “Action Plan for Three Groups of People in Programs of Poverty Alleviation Through Healthcare” proposes to implement EPHS and take the county as a unit to conduct a physical examination for eligible poverty people once a year. The project also requires doctors from township or county hospitals to sign contracts with poverty families to provide chronic disease management and health consultation [34]. Precious studies analyze the effects of the HPAP on health, education, and economic issues. The HPAP reduces the financial burden for the poverty population by decreasing out-of-pocket payments by 15% on average and the occurrence of catastrophic health expenditures. It also increases the number of annual hospitalizations [8]. Another economic vulnerability of poor households study shows an association between education and health poverty alleviation, which can cause synergistic effects in poverty alleviation [18]. The difference between economics and education are the main factors affecting health equity which poverty alleviation policy had a significant impact on [35]. Although our study shows the health outcomes differences between NPCs and PCs, it still takes more time to the effect of the HPAP.

For hypertension health management, this study shows that the difference in prevalence between PCs and NPCs is low, but the difference in standardized management prevalence is still high. In the “National Essential Public Health Services Specification (Third Edition)”, health management services for hypertensive patients require that patients with essential hypertension should provide at least 4 follow-ups each year which contain blood pressure measurement and lifestyle guide [13]. For these management details, the service quality in NPCs is higher than in PCs. Insufficient health resources and a lack of management service capacity may cause this gap between PCs and NPCs. The management service capacity of doctors in primary medical and health institutions in PCs needs to be improved. This study provides evidence of the effect of hypertension health management. Standardized hypertension health management is conducive to hypertension control in PCs and NPCs, and even non-standardized management works in NPCs but not PCs. Hypertension control in China is more dependent on primary medical institutions, and high-quality management is very effective for hypertension control. More than half of the hypertension patients under health management in PCs are not under standardized management, and low quality health management may not be effective in controlling hypertension. The quality of health management is as important as the development of management, and the management quality of PCs and NPCs needs to be further improved [36, 37]. Strengthening the EPHS, which includes hypertension and diabetes health management services, is one of the crucial measures for the HPAP for chronic disease control.

This study shows that the coexistence of multiple chronic diseases such as obesity, diabetes, and CKD among hypertension patients is another difficulty in hypertension control in PCs or NPCs. In the EPHS, health management for only hypertension or diabetes may be insufficient in PCs and NPCs. The HPAP requires formulating a unified and standardized chronic disease health management guidance. Under the direction of county hospitals, primary medical and health institutions arrange personalized health management according to the conditions of patients with chronic diseases. In order to control chronic disease in PCs, more measures for chronic disease comorbidity need to be implemented and sustained. In 2015, more than 44% of China’s poverty population were impoverished due to illness, but the number was almost reduced by half under the HPAP in 2018. A total of 15 million poverty people with infectious and chronic diseases had been treated and managed [38]. Although absolute poverty has been eliminated in China, there are still a large number of relatively poverty populations. In addition, the HPAP remain stable in the next five years as a transitional period to prevent people from returning to poverty due to illness and consolidate the achievements of poverty alleviation.

### Limitations

First, the survey data are cross-sectional, and causal relationships cannot be determined. Second, the survey data contains several self-reported variables, such as the diagnosed hypertension, physical examination, hypertension health management, and lifestyle influencing factors, which may cause bias and inaccuracy. Third, the survey data do not include personal poverty information, and the estimation of the situation of the poverty population may be biased. Fourth, due to the CCDRFS period, we can only evaluate the short-term health effect of the HPAP after two years of implementation.

## Conclusions

In summary, although hypertension prevalence in PCs is lower than NPCs, the proportion of physical examination, hypertension control and treatment prevalence, hypertension health management, and standardized management prevalence are higher in NPCs. The equity and accessibility gap of health resources still existed between PCs and NPCs under the influence of the HPAP. Our study provides evidence for the effect of hypertension health management on hypertension control in both PCs and NPCs. The quality of management services in PCs still needs to be improved. Our study shows the HPAP has achieved initial results, and further study is required to clarify the comprehensive effect of the HPAP.

## Data Availability

Individual participant data in our study will not be made available. Please contact jianceshi@ncncd.chinacdc.cn to get further detailed data access policy and procedure.

## Abbreviations

HPAP: Health poverty alleviation project
EPHS: Essential public health services
PCs: Poverty counties
NPCs: Non-poverty counties
CCDRFS: China Chronic Disease and Risk Factors Surveillance
BMI: Body-mass index
CKD: Chronic kidney disease
TC: Total cholesterol
HDL-C: High-density lipoprotein cholesterol
SBP: Systolic blood pressure
DBP: Diastolic blood pressure
FBG: Fasting blood glucose
